# Overview of the Composition of Whole Grains’ Phenolic Acids and Dietary Fibre and Their Effect on Chronic Non-Communicable Diseases

**DOI:** 10.3390/ijerph19053042

**Published:** 2022-03-05

**Authors:** Jabir Khan, Muhammad Zahoor Khan, Yulin Ma, Yantong Meng, Aroosa Mushtaq, Qun Shen, Yong Xue

**Affiliations:** 1 National Engineering and Technology Research Center for Fruits and Vegetables, College of Food Science and Nutritional Engineering, China Agricultural University, Beijing 100091, China; kjabir135@gmail.com (J.K.); mengyantong@cau.edu.cn (Y.M.); aroosamushtaq62@gmail.com (A.M.); shenqun@cau.edu.cn (Q.S.); 2 Key Laboratory of Plant Protein and Grain Processing, College of Food Science and Nutritional Engineering, China Agricultural University, Beijing 100091, China; 3 State Key Laboratory of Animal Nutrition, Beijing Engineering Technology Research Center of Raw Milk Quality and Safety Control, College of Animal Science and Technology, China Agricultural University, Beijing 100091, China; zahoorcau@cau.edu.cn (M.Z.K.); bs20193040395@cau.edu.cn (Y.M.); 4 Xinghua Industrial Research Centre for Food Science and Human Health, College of Food Science and Nutritional Engineering, China Agricultural University, Xinghua 225700, China

**Keywords:** whole grains, dietary fibres, phenolic acids, cardiovascular diseases (CVDs), obesity, type 2 diabetes (T2D), cancer

## Abstract

Chronic non-communicable diseases are the major cause of death globally. Whole grains are recommended in dietary guidelines worldwide due to increasing evidence that their consumption can improve health beyond just providing energy and nutrients. Epidemiological studies have suggested that the incorporation of whole grains, as part of a healthy diet, plays a key role in reducing one’s risk for cardiovascular diseases (CVDs), obesity, type 2 diabetes (T2D) and cancer. Phenolic acids and dietary fibre are important components found in whole grains that are largely responsible for these health advantages. Both phenolic acids and dietary fibre, which are predominantly present in the bran layer, are abundant in whole-grain cereals and pseudo-cereals. Several studies indicate that whole grain dietary fibre and phenolic acids are linked to health regulation. The main focus of this study is two-fold. First, we provide an overview of phenolic acids and dietary fibres found in whole grains (wheat, barley, oats, rice and buckwheat). Second, we review existing literature on the linkages between the consumption of whole grains and the development of the following chronic non-communicable diseases: CVDs, obesity, T2D and cancer. Altogether, scientific evidence that the intake of whole grains reduces the risk of certain chronic non-communicable disease is encouraging but not convincing. Based on previous studies, the current review encourages further research to cover the gap between the emerging science of whole grains and human health.

## 1. Introduction

Chronic non-communicable diseases are the major cause of mortality worldwide. According to a World Health Organization (WHO, Geneva, Switzerland) report, more than 15 million individuals between the ages of 30 and 69 die each year from chronic non-communicable diseases; 85% of these premature deaths occur in low- and middle-income countries [1]. Based on WHO reports for 2012 to 2016, consumption of whole grains may decrease the risk of non-communicable diseases (e.g., type 2 diabetes, cardiovascular diseases and obesity) [2]. The health aspects of whole grains have long been known. Grains play an integral role in most diets as they are the primary energy source. Wheat and rice are the most extensively consumed grains, whereas oats, barley, and buckwheat are low globally [3]. Some grains, such as wheat and rice, are usually consumed in refined form, produced after removing the outer layer. In addition, whole grains consist of intact ground, cracked or flaked kernels when the inedible parts are removed, such as hull and husk [4]. Thus, whole grain is composed of a starchy endosperm, the germ and the bran (including aleurone), with their relative proportions the same as those in the intact kernel. In contrast, refined grain products lack one or more components of the integral kernel. The bran and the germ are removed from the starchy endosperm in refined grains. The starchy endosperm alone accounts for 75–80% of the grain weight, and the bran contributes the rest, though the percentages vary between different grains and varieties. In addition, bran is the major component of phenolic acids, fibres and minerals in whole grains.

Epidemiological studies have shown that the intake of whole grains can reduce the risk of certain chronic non-communicable diseases, including obesity [5], cardiovascular diseases (CVDs) [6], type 2 diabetes (T2D) [7] and certain cancers [8]. In contrast, several studies on refined grains have shown a link between high refined grain intake and an increased risk of obesity [9], CVDs [10], diabetes [11,12,13] and cancers [14], while only a few studies have found null associations [15,16,17,18]. These studies support health recommendations to replace refined grains with whole grains. The health benefits of consuming whole grains may be attributed to the synergistic effects of the bran and germ components, which have inherently a higher dietary fibre content than refined grain products; the bioactivity of all nutrients; and the contributions of a wide range of phytochemicals in whole grains, such as phenolic acids/flavonoids, tocols, alkylresorcinols, avenanthramides and oryzanols [19,20,21,22]. In particular, phenolic acids have gained great attention because of their antioxidant, anti-inflammatory, and anti-carcinogenic activities [23,24]. Based on recently published studies, it has been suggested that phenolic acids and dietary fibre, coupled with whole grains, have numerous health benefits [20]. In addition, it has been well established that bran and germ fractions have positive health effects on both animals and humans via two mechanisms: first, by releasing indigestible fibres that influence gut microbiota composition and activity; second, by giving substrates such as resistant starch, non-starch polysaccharides (β-glucan and arabinoxylans) and phenolic acids that can be metabolised into useful metabolites of microbiota [25].

Thus, whole grain products, which contain more dietary fibres than refined grain products and often have a dietary fibre profile with a good balance of soluble and insoluble fibre components, affect human health [26]. In fact, the benefits of consuming whole grain cereals are connected to their higher fibre content and their content of fatty acids, vitamins, and other bioactive components [19,27]. It has been shown that phenolic acids and dietary fibres in whole grains have a significant effect on human health and provide protection against chronic non-communicable diseases [28,29,30].

According to already published data, the intake of whole grains can lower the risk of chronic non-communicable diseases. Therefore, there is general agreement that consuming whole grains may help in the prevention of several chronic non-communicable diseases. However, evidence from prospective cohort studies is sometimes mixed, as some individual publications have shown no significant or even contradictory findings. These considerations have increased researchers’ interest in investigating the influence of consuming whole grains on human health. Thus, the first aim of the present study was to discuss the composition of phenolic acids and dietary fibres in whole grains, as they are present in the most commonly consumed grains. Second, we discussed the effect of the consumption of whole grains on CVDs, obesity (the proposed mechanism and the high risk of obesity in relationship with other chronic diseases), T2D and cancers. Altogether, the scientific evidence that the intake of whole grains prevents the risk of certain chronic non-communicable diseases is encouraging but not convincing. Recent evidence suggests that whole grains’ phenolic acids and dietary fibres coupled with whole grains may be more beneficial healthwise than individual isolated components. Further studies are required to address this research gap in the association between the consumption of whole grains and the impact on human health.

## 2. Materials and Methods

In the present study, we have summarised and reviewed the phenolic acids and dietary fibres of whole grains. Then we examined existing literature on the association between the consumption of whole grains and the development of the following non-communicable chronic diseases: CVDs, obesity, T2D and cancer. All the materials for the current review were searched for in PubMed and Google Scholar, including human studies, such as observational (cross-sectional studies, case-control studies and cohort studies) and intervention studies. The major keywords used for the search of literature were whole grains, dietary fibres, phenolic acids, CVDs, obesity, T2D and cancer. In the present review, pertinent data published in the English language in reputable peer-reviewed journals have been included for discussion. However, all content available in the form of conference abstracts, books and unpublished findings were excluded.

## 3. The Phenolic Acids and Dietary Fibres in Whole Grains

### 3.1. Phenolic Acids in Whole Grains

Phenolic acids are found in a variety of cereals, legumes and other seeds, where they serve as a building material for cell wall matrices by establishing bridges with macromolecules, such as cellulose, hemicellulose and pectin, allowing for the formation of compact cell wall structures. The three forms of phenolic acids in grains include free, conjugated and bound forms [31,32]. According to the study of Adom and Liu [33], bound phenolic acids account for 70–95% of total phenolic acids via ester or ether linked to cell wall polysaccharides and cross links between them intramolecularly and/or intermolecularly to form networks. Consistently, it has been demonstrated that the bran/germ fraction contains 83% of the total phenolic content [34]. Phenolic acid is found mostly in the cortical layer of grains, where ferulic acid is the most abundant, followed by oxalic acid, p-coumaric acid and caffeic acid [32]. The quantity of phenolic compounds in whole-grain cereals varies depending on grain type, variety and portion [33,35,36]. Wholegrain phenolic acids are classified as hydroxybenzoic acids and hydroxycinnamic acid, respectively, based on their C1–C6 and C3–C6 skeletons. The difference among these derivatives is the type and number of functional groups substituted on the aromatic ring. p-Hydroxybenzoic, vanillic, gallic and syringic acids are hydroxybenzoic acid derivatives, whereas hydroxycinnamic acids include ferulic, p-coumaric, caffeic and sinapic acids [37] (Table 1), which are found as esters and glycosides. The most common phenolic acid compounds in whole grains are vanillic, ferulic acid caffeic, syringic and p-coumaric acids [38], which are distributed in large quantities in aleurone, embryo and pericarp but in far less quantities in the starchy endosperm of the cereal grains.

In Table 2, we have reviewed four hydroxybenzoic acids and four hydroxycinnamic acids found in whole grains. Hydroxybenzoic acids include *p*-hydroxybenzoic, gallic, vanillic and syringic acids. *p*-Hydroxybenzoic is found in oats, rice, buckwheat, wheat and barley [39]. The average range of *p*-hydroxybenzoic found in wheat is 7.5 μg/g and in barley is 110.7 μg/g. Gallic acid is distributed in wheat, barley, oats, rice [40] and buckwheat [39]. Among other grains, oats have the highest content (121.5 μg/g) while buckwheat has the lowest (36.8 μg/g). Vanillic acid is found in wheat, barley, oats, rice [40] and buckwheat [39]. The average content ranges from 7.2 μg/g in buckwheat to 19.6 μg/g in barley. Syringic acid has been reported in wheat [41], barley [42], oats [40], rice [43] and buckwheat [44], and its content was the highest in buckwheat, at 49.9 μg/g, and the lowest in barley, at 12.8 μg/g.

Hydroxycinnamic acids include ferulic, *p*-coumaric, caffeic and sinapic acids. Ferulic acid is omnipresent in plants and is derived from phenylalanine and tyrosine metabolism. Ferulic acid is found mostly in the cell walls of wheat [42], barley [40], oats, rice [47] and buckwheat [39,44]. The average content of ferulic acid in these grains ranges from 38.9 to 514.8 μg/g, with oats having the greatest amount and buckwheat having the lowest. Wheat, barley [42], oats and rice [40], as well as buckwheat [39], have all been found to contain *p*-coumaric acid. The average content of *p*-coumaric acid in these grains is 18.1 μg/g of dry weight in buckwheat and 607.3 μg/g of dry weight in oats. Wheat, barley, oats, rice [40] and buckwheat [39] all contain caffeic acid. The average caffeic acid contents range from 2.2 μg/g of dry weight in rice to 66.3 μg/g of dry weight in buckwheat. Sinapic acid can be found in a variety of plants, including wheat [45,46], barley, oats, rice [40,47] and buckwheat [39,44]. The average content in these grains ranges from 22.3 μg/g of dry weight in buckwheat and 62.7 μg/g of dry weight in oats.

As noted in Table 2, in the review of four hydroxybenzoic acids and four hydroxycinnamic acids in five whole grains, except for buckwheat, ferulic acid has the highest prevalence in all grains. Ferulic acid ranks second in oats, *p*-coumaric acid ranks first and caffeic acid ranks second in buckwheat, and the ranking becomes more variable in the rest of the phenolic acids found in cereals and pseudo-cereals. Oats, wheat, barley, rice and buckwheat are in descending order of the sum of the eight reviewed phenolic acids. Oats, wheat, barley, rice and buckwheat are in descending order of the four hydroxycinnamic acids; and wheat, buckwheat, barley, rice and oats are in descending order of the four hydroxybenzoic acids. According to these comparisons, each grain prefers one phenolic acid synthesis pathway over the others, leading to a unique phenolic acid profile. Due to low synthesis, buckwheat’s phenolic acid concentration, including *p*-hydroxybenzoic acid, caffeic acid and syringic acid, is substantially lower than in other grains.

### 3.2. Dietary Fibre in Whole Grains

According to Health Canada 2020, dietary fibres are defined as “carbohydrates with a degree of polymerisation of 3 or more that naturally occur in foods of plant origin and that are not digested and absorbed by the small intestine” [48]. Based on the water solubility of dietary fibre, they are classified into two types: insoluble dietary fibre (IDF) and soluble dietary fibre (SDF). Dietary fibre may be derived from different sources, such as cereals, fruits and vegetables. The quantity and composition of dietary fibre might differ depending on the source. Cereals are a good source of dietary fibre, and both soluble and insoluble dietary fibre help to decrease the risk of numerous chronic non-communicable diseases [49,50].

The total dietary fibre (TDF) content of both IDF and SDF of wheat ranges from 9 to about 20% (on a dry weight basis) [51] (Table 3). The cell walls of wheat’s starchy endosperm cells are made up of two primary types of dietary fibre components, i.e., arabinoxylan and β-glucan. In wheat grains, β-glucan and arabinoxylan generally account for about 20 to 70% of the total dietary fibre content. Small quantities of cellulose and glucomannan may also be found in these cell walls.

Oats and barley are excellent sources of IDF and SDF as well as other bioactive compounds. The IDF fraction is primarily found in the cereal’s bran, whereas the SDF fractions are found mostly in the endosperm cell walls [52]. On a dry matter basis, the TDF content of barley and oats varies from 10 to 28% [53] and 10 to 38% [54,55], respectively (Table 3). Both oats and barley contain β-glucan as the primary non-starch polysaccharide throughout the entire kernel; arabinoxylan is also found in both grains, although in considerably lower concentrations. In barley and oat cereals, β-glucan and arabinoxylan account for about 20 to 70% of the total dietary fibre content. Cereals’ β-glucan is composed of cellotriosyl and cellotetraosyl units, which are connected together by 1–3 linkages [56]. The concentration of β-glucan in oats and barley varies, depending on the genotype: in barley, β-glucan is distributed equally throughout the endosperm, but in oats, it is more concentrated in the outer layers of the endosperm [57]. Whole-grain barley has the same amount of β-glucan as oats. Barley varieties with low amylose content can even provide 1.5 to 4 times more β-glucan as compared to oats [56].

**Table 3:** The dietary fibre content of five whole grains (g/100 g).

Whole Grains	TDF	IDF	SDF	Reference
Wheat (*Triticum aestivum* L.; *Triticum durum Desf*.)	11.6–17.0	10.2–14.7	1.4–2.3	[58]
	10.2–15.7	7.2–11.4	0.9–2.9	[59]
	9.2	-	-	[60]
Barley (*Hordeum vulgare* L.)	14.6–27.1	-	-	[55]
	16.8–27.9	12.0–22.1	2.6–5.0	[61]
	10.1	-	-	[53]
Oats (*Avena sativa* L.)	13.7–30.1	-	11.5–20.0	[54]
	10.3	6.5	3.8	[62]
	11.5–37.7	8.6–33.9	2.9–3.8	[55]
Rice (*Oryza sativa* L.)	9.9	5.4	4.4	[63]
	2.7–4.9	1.9–4.2	0.6–1.1	[64]
Buckwheat (*Fagopyrumesculentum Moench*.)	7.0	2.2	4.8	[65]
	11.9	5.8	6.1	[66]

Note: TDF: total dietary fibre; IDF: insoluble dietary fibre; SDF: soluble dietary fibre.

The TDF level of rice varies from 2.7 to about 9.9%. This wide range of dietary fibre level is partly due to variations across rice varieties [65,67]. The authors found that brown rice has higher dietary fibre content than the content found in white rice, in which, essentially, the outer kernel layers have been removed by abrasive milling. The dietary fibres in rice kernel are mainly found in the hull and bran layer, the same as in other grains [68]. The major components of the IDF fraction in rice are cellulose and water-insoluble hemicellulose, whereas the SDF is made up of arabinoxylan and β-glucan [69].

The TDF content of buckwheat groats is 7–11.9%, which is lower than the dietary fibre content found in other cereals, such as wheat, barley and oats. The majority of dietary fibres from buckwheat groats (70%) are water insoluble [70]. Pectin, arabinogalactan and xyloglucan are the most common water-soluble fibres in buckwheat seeds [71]. The authors found pectin in the outer and inner epidermis of the cell walls as well as in the endosperm of buckwheat seeds.

It has been summarised in Table 3 that oats contain the highest dietary fibre levels, from 10 to 38%, while rice contains the lowest dietary fibre level among cereals (2–5%). The ranks become variable in the rest of the cereals. The descending order of the sum of total dietary fibre in Table 3 is oats, barley, wheat, buckwheat and rice. In a few studies, both soluble and insoluble dietary fibre in wheat and barley were undetected. The consumption of whole-grain fibre has been linked to a reduced risk of chronic non-communicable diseases. Diets rich in fibres are an important part of T2D management, since they enhance glycemic control, blood lipids, body weight and inflammation and reduce premature mortality. Further experimental trials are required to confirm the contents of TDF, IDF and SDF in different whole grains.

## 4. Linkage between Consumption of Whole Grains and the Development of Chronic Non-Communicable Diseases

The American Association of Cereals Chemist International (AACCI) issued a formal definition of whole grains in 1999 [4]: The “whole grain shall consist of intact, ground, cracked or flaked caryopsis (kernel), whose principle anatomical components including starchy endosperm, germ and bran are distributed in the same relative proportions as they exist in the intact caryopsis.” However, in 2006, the AACCI whole grains task force working group broadened the definition of whole grains to include pseudo-cereals [72] (Table 4). Pseudo-cereals were included because their overall macronutrient composition is comparable to that of cereals and they are used in the same traditional way as cereals. Furthermore, in recent years, whole grains have been the focus of significant scientific, governmental and commercial interest, as epidemiological studies have increasingly evaluated their defensive role against many chronic diseases, particularly those associated with chronic non-communicable diseases, such as CVDs and T2D [73,74].

Chronic non-communicable diseases (CVDs, obesity, T2D and cancer) are increasing rapidly worldwide [75]. Whole grains have numerous beneficial impacts on human health due to dietary fibres and phenolic compounds, which have been found to be associated with a reduced risk of chronic non-communicable diseases [20,76]. Several studies have suggested that people who consume three or more servings of whole grains/day have a 20–30% lower risk of diseases than those who consume a small amount of whole grains and this level of protection is not observed with the consumption of refined cereals [70,77,78]. A health-tracking study of professionals monitored 42,898 males and found that those who consumed approximately three servings of whole-grain cereals per day had a 37% lower chance of developing T2D [79]. The data brought together in prospective cohort studies show that the intake of whole grains reduces the relative risk of T2D by 30% [80]. Another study found that, compared to refined grains, the consumption of whole grains is inversely related to obesity [81]. A study based on 16 cohorts from seven countries demonstrated that body mass index is negatively associated with whole-grain dietary fibre intake [82]. Similarly, another study [83] found an inverse relationship between the consumption of whole grains and the risk of being overweight or obese. The authors found that the association in the male participants was stronger than that in the female participants. Furthermore, the consumption of whole grains has been linked to a lower risk of some cancers, including colorectal cancer [15], mouth/throat cancer and upper digestive tract cancer [84]. As a result, multiple epidemiological investigations, notably large prospective studies with millions of people followed for years, have discovered an inverse link between a whole-grain diet, including bran, and the risk of chronic non-communicable diseases [74,85,86]. In addition, research strongly supports the intake of whole grains’ phenolic acids with a decreased risk of certain chronic non-communicable diseases [29,30,70]. Furthermore, in a 12-week randomised double-blind placebo-controlled study, it was documented that whole-grain dietary fibre may with phenolic acids have a protective effect [87]. It shows that dietary fibre and phenolic acids in whole grains have associations with an improved health status.

### 4.1. The Effect of Consumption of Whole Grains on CVDs

Cardiovascular diseases are the world’s leading cause of death. According to the World Health Organization, 17.9 million people died from CVDs in 2019, representing 32% of all global deaths; CVDs were responsible for 85% of these deaths [88]. Researchers predict that by 2030, chronic non-communicable diseases will account for more than three-quarters of global deaths; CVDs alone will account for more deaths in low-income countries than other diseases [89].

A study tracking health professionals [19] examined the consumption of whole-grain cereals, bran and germ in terms of CVD risk using the data on food consumption frequency. The authors found that the added germ has no link with CVD risk. Based on the above-published data, the link between consuming whole-grain cereals and decreased CVD risk is obvious, indicating that the bran of whole-grain cereals might be a significant component in this relationship.

A systematic review has documented a significant inverse relationship between the consumption of whole grains and the risk of CVDs, cancer and other causes of specific mortality [90]. In addition, a cohort study in a Spanish working population evaluated the relationship between dietary fibre type and CVD risk factors [91]. When analysing the blood sample, the above study found a negative association between insoluble dietary fibre consumption and total cholesterol and blood pressure. In contrast, soluble dietary fibre intake had an inverse relationship with triglyceride content. Cereals’ β-glucan is one of the most common types of soluble dietary fibres that affect CVD risk. After reviewing the scientific data, the Food and Drug Administration approved a health claim that soluble fibres from whole grains may lower the risk of heart diseases [92]. These findings support the advice that people eat more whole grains to improve their health.

Research on whole-grain bran shows that *p*-coumaric acids have free radical scavenging properties, potentially protecting against CVDs because of their ability to decrease the low-density lipoproteins’ resistance to cholesterol oxidation [93]. A meta-analysis including 24 clinical studies [94] concluded that the consumption of whole grains lowers low-density-lipoprotein cholesterol and total cholesterol and tends to lower triglycerides compared with non-wholegrain control diets but has no effect on high-density-lipoprotein cholesterol. Interestingly, they did not find a threshold dose or dose-dependent association. Another meta-analysis study, which covered 45 prospective cohort studies and 21 randomised controlled trials, compared the consumption of whole grains to those who never or seldom consumed whole grains and found that those who consumed 48–80 g of whole grains/day had an ~26% lower risk of T2D and an ~21% lower risk of CVDs [84].

The consumption of whole grains may reduce the risk of CVDs associated with different parts of the cereal grain due to differences in the composition and constituents of phenolic acids and dietary fibres. Whole grains contain the endosperm, the bran layer and the germ, whereas refined grains contain only the endosperm. The bran layer and the germ are high in dietary fibres, polyphenols and other components that may provide cardiovascular protection. Based on the above studies, the health benefits of whole grains’ consumption are thought to be associated with fibres and phenolic acid, which is mainly in the bran and germ fraction of the whole grains [95]. Although the processes underlying these effects are not fully understood, they are likely to be closely related to the antioxidant activity of whole grains [96,97]. Future prospective studies may address the question of whether the intake of whole grains is directly related to CVDs and whether the associations are primarily driven by phenolic acids, dietary fibres or some other related aspect of the diet.

### 4.2. The Effect of Consuming Whole Grains on Obesity

Obesity is a serious health concern in developed countries, and it has been associated with a wide range of metabolic diseases, CVDs, T2D and several types of cancer [98]. Worldwide, obesity is currently the most common metabolic disease, although the prevalence varies widely among different countries. The increase in obesity is defined as a surplus amount of body fat and is confined to affluent societies and developing countries. Epidemiological studies have indicated that regular use of whole grains leads to a lower risk of developing obesity [7]. A cross-cultural study of 16 cohorts from seven countries discovered that the body mass index (BMI) and the subscapular skin fold thickness were negatively associated with total dietary fibre consumption, suggesting that reducing fibre intake is the key factor in body fat accumulation [99]. Furthermore, a cohort study conducted in the Netherlands documented an inverse relationship between whole grains’ intake levels and the incidence of overweight or obesity in both men and women, and the correlation in men was stronger than in female participants [9]. Over a 12-year follow-up period, a large prospective study on 74,091 females indicated that the consumption of whole grains and bran when combined with body weight measurements reduced the risk of obesity and weight gain by 19% and 23%, respectively [100]. In another prospective study, compared with the lowest consumption, the highest consumption of whole grains and bran led to a 23% lower risk of weight gain in an over 8-year follow-up period in 26,082 males [101]. According to a short-term study, the consumption of soluble dietary fibre β-glucan can improve the postprandial satiety feeling and reduce body weight and the intake of total calories [102]. The authors found that consuming β-glucan from cereal sources significantly reduces body weight. The gel-forming ability of soluble-glucan and other soluble fibres, as well as the bulking influence of insoluble fibres, may be connected with prolonged satiety feelings [103]. Similarly, in long-term prospective observational studies, the intake amount of whole grains on a daily basis may contribute to a smaller waist circumference, a lower BMI (weight in relation to height) and lower body fat levels [104,105]. The evidence from randomised controlled research demonstrates variations in the benefits of a whole-grain food diet in terms of body weight compared to control (non-wholegrain meals). Larger and longer-term human intervention trials are necessary to determine if whole grains contribute solely to a healthier lifestyle status.

Furthermore, in a small prospective study, it was documented that those who consumed the highest amount of whole grains and bran showed a 7.2% reduction in obesity during the 2-year follow-up period [106]. The epidemiological study also showed that whole grains are inversely correlated with a reduced risk of obesity [107]. These studies show consistent inverse correlations between the intake of whole grains and bran and BMI, weight gain, body weight and the risk of obesity. Despite consistent inverse correlations, there were no significant changes in absolute body weight or weight gain between the highest- and lowest-consumption groups in these prospective trials. The results from the cohort study performed by [106] are similar to data reported in adults [107]. The synergistic effect of several whole-grain dietary fibre components with phenolic acids may be involved in the protective mechanism against and obesity [19,20,28,29,30].

Several factors might explain the effect of whole grains on body weight management. Whole-grain foods may enhance satiation due to their high volume, low energy density and lower palatability. Furthermore, whole grains may improve satiety (delayed return of appetite after a meal) for up to several hours after a meal. Grains high in soluble fibres (such as oats and barley) enhance intraluminal viscosity, prolong gastric emptying and inhibit nutrient absorption in the small intestine. Although preliminary evidence suggests that whole grains may influence body weight regulation, more research is needed to determine the independent effects of whole-grain bran, dietary fibre and phenolic acids on obesity, as well as epidemiological studies and clinical trials, to confirm health benefits.

### 4.3. Relationship of High Risk of Obesity with Other Chronic Diseases

Scientific studies support the consumption of whole grains to reduce the risk of being overweight and obese [7,25,75,81]. The major impact of the intake of whole grains may be a reduced appetite and a longer sensation of fullness [108]. The intake of whole-grain meals, the particle size and the structural integrity alter the quantity of chewing necessary for ingestion of whole-grain foods. Increased chewing may induce satiation by enhancing gastric distention, improving gut hormone responses [83,109] or slowing the eating rate [110,111]. Whole-grain foods have a lower energy density, which is defined as digestible energy per unit weight, than refined grain foods. The low digestible energy per unit mass and many dietary fibres’ water-holding capabilities may contribute to this effect [112]. According to short-term studies, individuals tend to eat a consistent weight of food regardless of calorie content, showing that the mass of food ingested influences hunger more than the quantity of energy consumed [113]. As a result, lowering dietary energy density reduces energy intake without an increase in appetite [114].

**Figure 1:**
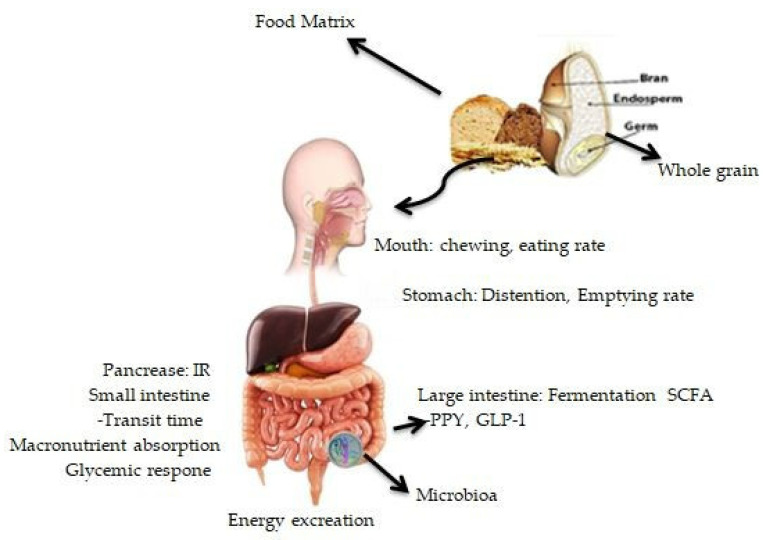
The influence of whole grains on physiologic parameters regulating body weight and composition is mediated by the structural and physicochemical features of whole-grain meals. IR: insulin response; GLP-1: glucagon-like peptide-1; PYY: peptide; SCFA: short-chain fatty acid.

Furthermore, studies have highlighted the possible role of gut microbiota in modulating correlations between the consumption of whole grains and body weight management [115,116,117]. Researchers have discovered that short-chain fatty acids (SCFAs) synthesised during the fermentation of specific fibres within whole grains can help to regulate body weight and composition by acting as metabolisable energy sources. In addition, SCFAs directly regulate the hepatic and peripheral glucose and lipid oxidation and stimulate the secretion of the gut hormones peptide-YY and glucagon-like peptide 1 (GLP-1), which suppress appetite, slow gastrointestinal transit and alter glucose metabolism [101,118] (Figure 1). A number of variables influence SCFA synthesis, including the composition of the gut microbiota and the availability of a fermentable substrate [119]. The prebiotic effect is the symbiotic relationship between the gut microbiota and the human host in which specific fermentable carbohydrates selectively promote the production of colonic bacteria beneficial to host health [120], demonstrating the relationship between substrate availability and gut microbiota composition. Emerging data suggest that the gut microbiota is connected to human obesity [120,121,122,123] and is responsive to several dietary variables [124], suggesting a possible function for whole grains in body weight management via gut microbiome modulation. Though these studies provide some support for whole-grain components having a beneficial effect on body weight control, additional research is needed to discover whether eating smaller doses of whole grains would have a similar beneficial effect.

### 4.4. Relationship of Obesity with Other Chronic Diseases

**Figure 2:**
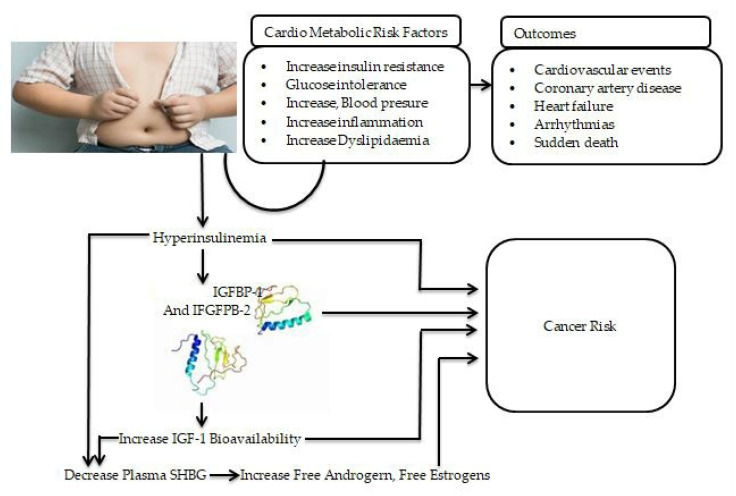
The link between obesity and other chronic non-communicable diseases and proposed mechanisms of obesity and increased cancer risk. IGF-1: insulin-like growth factor-1; IGFBP-2: insulin-like growth factor binding protein-2; IGF-1: insulin-like growth factor -1; SHBG: sex hormone-binding globulin.

Obesity has been discovered to have a substantial relationship with insulin resistance and T2D. In obese patients, substances such as glycerol, non-esterified fatty acids and cytokines markers are associated with the growth of insulin resistance. In severe T2D, the pathogenesis of T2D involves the weakening of pancreatic beta-islet cells or insulin resistance or both [125]. Insulin resistance is the underlying cause of both T2D mellitus and obesity. Insulin sensitivity normally changes during the life cycle, as shown during pregnancy, puberty and the ageing process [125]. Furthermore, lifestyle modifications, such as increased carbohydrate intake and increased physical activity, are variables that contribute to insulin sensitivity [126]. In addition, intra-abdominal fat is more important in insulin resistance because it is more lipolytic and does not readily respond to anti-lipolytic insulin action [127]. CVDs are connected to T2D and obesity because of the correlation of inflammation (low grade), as demonstrated in Figure 2. The over-expression of cytokines, such as interleukin-6 (IL-6), IL-1, leptin, plasminogen activator inhibitor-1 (PAI-1), resistin monocyte chemo attractant protein-1 (MCP-1), angiotensin and tumor necrosis factor-α (TNF-α) fibrinogen, causes inflammation and lipid accumulation, which has a disastrous effect on blood vessels and can eventually cause endothelial dysfunction, cardiomyopathy and myocardial infarction [128,129,130,131]. The over-expression of these cytokines is linked to the connection between insulin resistance and T2D. However, considering whole grains’ nutritional advantage over refined grains, whole grains should be recommended as part of a health-promoting diet.

### 4.5. Proposed Mechanism of High Risk of Obesity and Cancer Risk

In addition to environmental factors and genetic susceptibility, multiple mechanisms have been proposed to explain the epidemiologic links between obesity and cancer [132,133,134]. Obesity, especially central/visceral obesity, results in insulin resistance and prolonged compensatory hyperinsulinemia. Increased insulin levels have been shown to induce mitogenic effects and lead to cancer risk by activating both the insulin receptor and the insulin-like growth factor 1 (IGF-1) receptor. Hyperinsulinemia can also decrease the production of insulin-like growth factor-binding protein 1 (IGFBP-1) in the liver and other organs and is linked to lower plasma insulin-like growth factor binding protein-2 (IGFBP-2) levels. This reduction in IGFBP-1 and IGFBP-2 levels, in turn, increases IGF-1 bioavailability, which stimulates cellular proliferation and prevents apoptosis via its receptor in various organs [135,136,137,138] (Figure 2). Increased estrogen and androgen levels are also known to play a role in the development of cancer, especially in endometrial and postmenopausal breast malignancies. Circulating sex hormone-binding globulin levels have much lower production in the liver. Insulin resistance and persistent compensatory hyperinsulinemia appear to be important in the pathophysiology of obesity-related carcinogenesis, which may differ depending on the cancer type in individuals with central obesity and hyperinsulinemia.

### 4.6. The Effect of Consuming Whole Grains on T2D

Worldwide, T2D is a key health problem and carries a socioeconomic burden, especially in low- and middle-income countries [139,140]. Globally, the incidence of T2D was predicted to reach 439 million by 2030 [141]. According to the authors, there will be a 69% increase in the number of adults with T2D in developing countries and a 20% increase in developed countries between 2010 and 2030. Wholegrain cereals’ consumption is related to a lower incidence of T2D [6,73,75,79,80,88,131]. In long-term studies of almost 90,000 women [142] and nearly 45,000 men [143], it has been documented that the intake of whole grains can lower the risk of developing T2D, when combined data from both studies show an approximately 30% lower risk of developing T2D found in those who used more whole-grain dietary fibres compared to others. According to another study, individuals who consumed large quantities of refined grains and small amounts of whole grains had a 57% higher risk of T2D than those who consumed large amounts of whole grains [79]. Furthermore, a health professional follow-up study that followed 42,898 men reported a 37% lower risk of T2D linked to about three servings of whole grains per day [80]. When these data were combined together in prospective cohort studies, they indicated that the consumption of whole-grain cereals reduces the relative risk of T2D by 30%. Randomised, controlled dietary trials in people and other experimental research show a causal link between the intake of whole grains and T2D prevention [144,145]. In addition, a study found that a diet high in whole-grain products lowers the postprandial insulin and plasma triglyceride levels in people with metabolic syndrome by 29% and 43%, respectively [146]. The researchers found that the effects of whole-grain cereals on postprandial insulin and plasma triglyceride concentrations might explain the relationship between the consumption of cereals and a reduced risk of T2D and CVDs. However, researchers have found largely contrasting results in this field, clearly highlighting the need for more studies [84,147]. While the health benefits of consuming whole grains are most likely connected to the dietary fibres and phenolic acids found in whole-grain bran [22,28,29,30], additional study is needed to understand the effects deeply.

The phenolic acid in whole grains is a key contributor to overall antioxidant capacity and lowers the risk of chronic diseases [148]. Therefore, dietary components, such as phenolic acids, that decrease the risk of chronic diseases and a limited number of micronutrients that act as antioxidants may prevent the progression of metabolic syndrome and T2D by lowering oxidative stress [149]. In addition to their antioxidant capabilities, several cereals’ phenolic compounds have potential anti-inflammatory properties [150] and may thus influence T2D risk via this mechanism as well [151,152,153]. Although not elucidated, in clinical trials and epidemiological studies, the consumption of whole-grain bran has been linked with a reduced risk of T2D, probably due to the phenolic acids and fibres which are embedded in the bran [154]. In addition, the high nutritional and fibre contents in general and the physical structure of whole grains are thought to be the major cause of T2D [155,156]. Furthermore, one cohort study reported a negative association between total fibre consumption, particularly cereal fibres, and the incidence of T2D, and found that the fibre derived from fruits or vegetables does not influence the risk [86]. Adjustment for cereal fibre significantly decreased the connection between the consumption of whole grains and T2D risks, indicating that the relation may be due to cereals’ fibre or factors correlated with the intake of the cereals’ fibre.

The global prevalence of T2D has become a serious threat to human health in both developing and developed countries. Diet modification is one of the most significant factors in lowering the risk and controlling the development of diabetes complications. Scientific data suggest that the intake of whole grains regularly may reduce the incidence of chronic non-communicable diseases such as T2D. Because the whole-grain bran layer contains a wide range of functional components, including dietary phenolic acids and dietary fibres, it is important to determine which of these components may have the most protective benefit. We found a convincing inverse relationship between the consumption of whole grains and T2D, which is consistent with previous research. Clinical and epidemiological studies support the association of whole grains, phenolic acids and dietary fibres with a lower risk of disease. However, researchers have found largely contrasting results in this field, clearly highlighting the need for future research. We recommend more research on the independent effects of bran, phenolic acids and dietary fibres on T2D to cover the gap in the link between whole grains and T2D.

### 4.7. The Effect of Consuming Whole Grains on Cancer

According to the World Health Organization report 2021, cancer accounted for over 10 million deaths by 2020 and is considered a leading cause of death worldwide [157]. Whole-grain cereals’ intake has been found to be associated with a lower risk of cancer in numerous studies. Several studies have found that there is strong evidence of a link between the intake of whole grains and a lower risk of cancer diseases [8,48,55,147]. The consumption of whole grains may prevent cancer owing to intestinal microbiota, synthesis of short-chain fatty acids, reduced transit time, prevention of insulin resistance and antioxidant activity of phenolic acids, which protect by binding carcinogens and modulating glycemic response. This antioxidant effect is due to their phenolic acids, which alleviate oxidative stress [158]. The phenolic acids in the whole grain affect the cellular signal transduction pathways and hence influence cancer cell behavior, such as proliferation, apoptosis and invasion [159]. Bran antioxidants may contribute to cellular protection while also reducing oxidative damage. The ability of phenolic acids to prevent cancer has been linked to their ability to reduce oxidative damage to cells and cell components [160,161].

A meta-analysis of six trials on whole grains [94] found that every three servings (90 g/day) of whole grains can reduce the risk of colorectal cancer by 17%. A review of 40 studies on gastrointestinal cancer [162] revealed that those who consumed high amounts of whole grains had a reduction in cancer risk from 21% compared to 43% in subjects with low consumption. A meta-analysis study of over 786,000 individuals combining results from studies conducted in the U.S., the U.K. and Scandinavian countries [163] concluded that people who consumed 70 g of whole grains/day had a 22% lower risk of total mortality, a 23% lower risk of CVD mortality and a 20% lower risk of cancer mortality. However, a study of 58,279 males [164] showed no link between dietary fibre and colorectal cancer. Cohort studies have indicated a reduction in the incidence of specific cancers, such as colorectal in women [15], mouth/throat and the upper digestive tract cancer [84] and endometrial cancer [165]. In another cohort study, it was found that consuming more whole grains can reduce the incidence of colorectal cancer in women by 19% [166]. Furthermore, the prospective National Institutes of Health-AARP Diet and Health Study following 291,988 men and 197,623 women reported that the consumption of whole grains could reduce colorectal cancer risk by 21% [167]. As longitudinal studies are necessary to investigate cancer formation and progression, clinical evidence of the influence of whole grains or their contents on cancer risk cannot be readily established. Despite lacking support from clinical studies, preclinical studies provide a strong biochemical and molecular mechanism for the anti-carcinogenic activity of whole grains’ phenolic acids. For example, both ferulic acid and *p*-coumaric acid were found to be associated with the inhibition of cell growth by modulating cell cycle phases in colonic cancer cells [168]. A preclinical study [119] used human lung and colon adenocarcinoma cancer cell lines to demonstrate that caffeic acid, ferulic acid or *p*-coumaric acid inhibits cell adhesion and migration, which are important mechanisms in tumour metastasis. These preliminary findings indicate the health benefits of phenolic acids in whole grains in cancer prevention and protection.

Furthermore, free phenolic acids are more easily digested than bound phenolic acids in the upper digestive tract [169]. Because it is more difficult to digest the cell walls in bound phenolic acids, the digestion and absorption process takes place mostly in the large intestine. Bound phenolic acids are released from the cell wall in the large intestine in the form of glycosidic ligand by the activity of bacteria or similar enzymes and then reformed into glucoside, which is used by the human body via the glucose transporter in the cell. Consistently, [170] discovered that the interaction of phenolic acids with bacteria in the large intestine greatly decreases the risk of colon cancer and enhances the intestinal microbial environment. In addition, it was found that whole grains’ consumption relates inversely to colorectal cancer but the effect is small [171]. A meta-analysis of observational studies investigated the potential role of the consumption of whole grains in reducing the risk of pancreatic cancer [172]. The researchers concluded that a high intake of whole grains might lower the risk of pancreatic cancer. However, more cohort and prospective studies are needed to identify a stronger association.

Meta-analysis and observational studies have found an inverse relationship between the consumption of whole grains and different forms of cancer; longitudinal studies are necessary to examine cancer development and progression. Whole grains’ phenolic acids, dietary fibre and essential micronutrients were responsible for the observed protection. Several mechanisms have been proposed for the action of whole grains in terms of cancer, fermentation in the colon, contribution to reduced intestinal transit and improved intestinal health. Cereals also include antioxidants, which can protect against oxidative damage, playing a significant role in cancer development. Other bioactive compounds included in whole-grain cereals may influence hormonal levels and probably hormone-dependent cancers. Furthermore, the consumption of whole grains has the ability to lower insulin levels, which may be an indirect way of lowering cancer risk, given that several epidemiological studies have indicated that higher levels of insulin are associated with a greater risk of colon, breast and possibly other types of cancer. An indirect mechanism of protection may be a lower risk of obesity linked with higher consumption of whole grains, which is considered a significant risk factor for different forms of cancers. The link between the consumption of whole grains and other forms of cancers, such as breast, pancreatic, oral and pharyngeal cancer, is less studied and results are often conflicting. In addition, attention should be paid to the exact possible mechanism and the independent effect of whole-grain bran phenolic acids and dietary fibres.

## 5. Conclusions

Whole grains are rich in many components, including phenolic compounds and dietary fibres, which have been linked to the reduced risk of CVDs, obesity, T2D and cancers. The majority of health-associated components are concentrated in the bran and the germ, which are removed during the grain-refining process. Emerging evidence suggests that the intake of whole grains has benefits beyond providing basic nutrition, a fact sustained by epidemiological studies, which indicate a protective role of a whole-grain diet against obesity, CVDs, T2D and cancer. Several findings indicate those health effects may be due to the enrichment of phenolic acids with dietary fibres in whole grains. Because whole grains contain a wide range of dietary fibre, phenolic acids and other functional components, it is important to figure out which of these components may have the greatest protective effect against specific diseases. The role of the consumption of whole grains in disease prevention is promising but not conclusive, and more clinical trials and epidemiologic studies are needed. Future studies may address whether the whole grain’s bran and germ are directly associated with reducing the risk of obesity CVDs, T2D and cancers or whether the associations are primarily driven by dietary fibres, specific polyphenols or some other related aspect of the diet and could significantly contribute to the next generation of healthy cereal-based products. Thus, it will be of interest to ascertain the independent effects of bran, germ and phenolic acids and different types of fibres on chronic non-communicable diseases.

## Figures and Tables

**Table 1:** Chemical structure of four hydroxybenzoic acids (a) and four hydroxycinnamic acids (b).

**(a) Hydroxybenzoic Acids**			
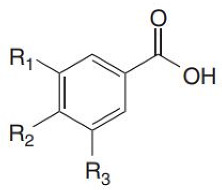			
	$R_{1}$	$R_{2}$	$R_{3}$
*p*-Hydroxybenzoic acid	H	OH	H
Gallic acid	OH	OH	OH
Vanillic acid	H	OH	${\mathrm{OCH}}_{3}$
Syringic acid	${\mathrm{OCH}}_{3}$	OH	${\mathrm{OCH}}_{3}$
**(b) Hydroxycinnamic Acids**			
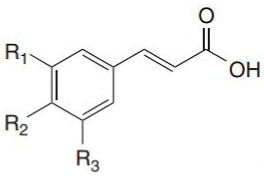			
	$R_{1}$	$R_{2}$	$R_{3}$
Ferulic acid	${\mathrm{OCH}}_{3}$	H	H
*p*-Coumaric acid	H	OH	H
Caffeic acid	OH	OH	OH
Sinapic acid	${\mathrm{OCH}}_{3}$	OH	${\mathrm{OCH}}_{3}$

**Table 2:** Review of four hydroxybenzoic acids and four hydroxycinnamic acids in five whole grains.

**Contents of Four Hydroxybenzoic Acids in Whole Grains (μg/g of Dry Weight)**					
Grains	*p*-Hydroxybenzoic acid	Gallic acid	Vanillic acid	Syringic acid	References
Wheat	7.5 (4.9–10.8)	89.2 (6.5–195.0)	15.4 (3.4–57.0)	19.5 (4.0–58.5)	[40,41]
Barley	110.7 (6.4–215.0)	82.6 (6.5–158.6)	19.6 (5.9–49.3)	12.8 (6.0–55.2)	[40,42]
Oat	12.0 (8.1–16.0)	121.5 (1.7–241.2)	16.3 (11.4–20.5)	19.2 (17.9–20.0)	[40]
Rice	25.7 (5.1–46.3)	51.7 (5.5–115.6)	19.2 (4.4–38.1)	52.3 (2.8–103.9)	[40,43]
Buckwheat	64.8 (19.6–110.0)	36.8 (26–71.0)	7.2 (1.2–15.0)	49.9 (36.3–63.5)	[39,44]
**Contents of Four Hydroxycinnamic Acids in Whole Grains (μg/g of Dry Weight)**					
	Ferulic acid	*p*-Coumaric acid	Caffeic acid	Sinapic acid	
Wheat	485.0 (11.6–870.0)	54.4 (3.5–523.0)	26.2 (0.5–51.9)	59.3 (22.4–157.8)	[40,42,45,46]
Barley	381.8 (155.1–601.9)	82.2 (18.4–151.4)	13.8 (5.6–21.9)	54.3 (18.8–43.5)	[40,42,47]
Oat	514.8 (249.4–1044.9)	607.3	6.4 (3.6–9.2)	62.7 (51.9–107.1)	[40,47]
Rice	219.7 (68.2–554.7)	45.3 (22.8–85.0)	2.2 (1.0–3.5)	58.7 (24.2–47.2)	[40,47]
Buckwheat	38.9 (4.4–122.8)	18.1 (1.7–37.7)	66.3 (8.0–105.9)	22.3 (2.2–37.7)	[39,44]

**Table 4:** Botanical names of whole grains used in the study.

Cereal Type	Botanical Name
Wheat	*Triticum* spp.
Barley	*Hordeum* spp.
Oats	*Avena* spp.
Rice	*Oryza* spp.
**Pseudo-Cereal**	**Botanical Name**
Buckwheat	*Fagopyrum* spp.

## Data Availability

All the data are already provided in the main manuscript.
